# Zinc-aspirin preconditioning reduces endothelial damage of arterial grafts in a rodent model of revascularization

**DOI:** 10.3389/fcvm.2023.1288128

**Published:** 2024-01-04

**Authors:** Kálmán Benke, Roland Stengl, Klára Aliz Stark, Yang Bai, Tamás Radovits, Sivakkanan Loganathan, Sevil Korkmaz-Icöz, Máté Csonka, Matthias Karck, Gábor Szabó, Gábor Veres

**Affiliations:** ^1^ Department of Cardiac Surgery, Martin Luther University Halle-Wittenberg, Halle (Saale), Germany; ^2^ Heart and Vascular Center, Semmelweis University, Budapest, Hungary; ^3^ Department of Cardiac Surgery, University of Heidelberg, Heidelberg, Germany

**Keywords:** CABG, zinc-aspirin, endothelium, ischemia-reperfusion injury, free graft

## Abstract

**Introduction:**

Coronary artery bypass grafting (CABG) is the most common cardiac surgical procedure. The prognosis of revascularization via CABG is determined by the patency of the used grafts, for which an intact endothelium is essential. The degree of ischemia-reperfusion injury (IRI), which occurs during the harvest and implantation of the grafts, is an important determinant of graft patency. Preconditioning with aspirin, a nonsteroidal anti-inflammatory drug has been shown to reduce the functional and molecular damage of arterial grafts in a rodent model. Studies have found that the zinc-aspirin complex may be able to exert an even better protective effect in pathological cardiovascular conditions. Thus, our aim was to characterize the protective effect of zinc-aspirin complex on free arterial grafts in a rodent model of revascularization.

**Methods:**

Donor Lewis rats were treated with either zinc-aspirin, aspirin, or placebo (*n* = 8) for 5 days, then the aortic arches were harvested and stored in cold preservation solution and implanted heterotopically in the abdominal cavity of the recipient rats, followed by 2 h of reperfusion. There was also a non-ischemia-reperfusion control group (*n* = 8). Functional measurements using organ bath and histomorphological changes using immunohistochemistry were analyzed.

**Results:**

The endothelium dependent maximal vasorelaxation was improved (non-transplanted control group: 82% ± 3%, transplanted control group: 14% ± 2%, aspirin group: 31% ± 4%, zinc-aspirin group: 52% ± 4%), the nitro-oxidative stress and cell apoptosis decreased, and significant endothelial protection was shown in the groups preconditioned with aspirin or zinc-aspirin. However, zinc-aspirin proved to be more effective in the reduction of IRI, than aspirin alone.

**Discussion:**

Preconditioning with zinc-aspirin could be a promising way to protect the function and structural integrity of free arterial grafts, thus improving the outcomes of CABG.

## 1 Introduction

Coronary artery bypass grafting (CABG) is a durable treatment for coronary artery disease (CAD) and it is the most frequently performed cardiac surgical procedure worldwide (1). The prognosis of revascularization via CABG is determined by the patency of the applied grafts. Arterial grafts have superior patency rates compared to vein grafts, and the use of the internal thoracic artery (ITA) leads to the most favorable outcomes (2). Graft failure is mainly due to acute thrombosis in the early phase after revascularization, and atherosclerosis in the long-term. Endothelial damage and dysfunction play a crucial role in both events, thus an intact endothelium is necessary in reaching satisfactory results after CABG (2). One of the most important endothelial damage causing factors is ischemia-reperfusion injury (IRI), which occurs during harvest, storage and implantation of free grafts (3). IRI is triggered by the restoration of blood flow after a prolonged ischemic period. Processes initiated by ischemia intensify during reperfusion, causing the release of various reactive oxygen species (ROS) through proinflammatory cytokines (i.e., TNF-α and IL-6) and increased endothelial interaction with circulating cells (leukocytes, platelets), leading to endothelial damage (4). The importance of IRI in free grafts is emphasized by the findings that the patency of free ITA grafts is remarkably lower than that of *in situ* ITA grafts (5).

In the clinical practice, free grafts are mostly stored in cold physiological saline or heparinized blood solutions after being harvested in order to preserve the endothelium. However, it has been shown that these solutions are not able to prevent the endothelial damage suffered by the grafts during storage and reperfusion (6). Thus, there is a need for effective options to preserve the integrity of the endothelium of bypass grafts. To achieve this, some promising storage solutions (3, 7) as well as drugs for pharmacological conditioning (8, 9) have been identified in preclinical studies. Our research group has shown the protective effect of aspirin preconditioning on the structural and functional integrity of free arterial grafts in a rodent model of arterial revascularization (10).

Aspirin (Asp) is a nonsteroidal anti-inflammatory drug with antithrombotic and antipyretic properties in addition to its anti-inflammatory effects. Asp blocks the cyclooxigenase-1 enzyme that results in the suppression of the platelet agonist and vasoconstrictor thromboxane, leading to platelet inhibition. The anti-inflammatory effect is achieved by the blockade of the cyclooxigenase-2 enzyme (7, 11). Due to these beneficial mechanisms, Asp plays an important role in the management of CAD patients, however, its administration prior to CABG is still a subject of debate (7).

Zinc (Zn) is a trace element with several physiological functions. It also possesses anti-inflammatory and antioxidant properties and has been found to protect various organs from IRI (12).

Experimental *in vitro* studies have shown that the combined Asp and Zn into Zn(Asp)_2_ (bis(aspirinato)zinc(II)) complex exert a more pronounced protective effect against ischemic damage and endothelial injury than treatment with aspirin alone (13, 14). We hypothesize that pretreatment with zinc-aspirin before bypass surgery can protect arterial free grafts from functional and morphological damage to a better extent than aspirin alone, thereby providing a promising option to improve the short and long-term outcomes of CABG. Thus, our aim was to study the potential benefits of pharmacological preconditioning with zinc-aspirin in a rat model of arterial revascularization.

## 2 Materials and methods

### 2.1 Animals

Male Lewis rats (250–300 g; Janvier Labs, Saint-Berthevin Cedex, France) were housed in standard cages in a room at a constant temperature of 22 ± 2°C under 12-h light/dark cycles and fed on standard laboratory rat diet and had access to water *ad libitum*. The rats were acclimatized for at least 1 week before the experiments. All animals received human care according to the “Principles of Laboratory Animal Care” drafted by the National Society for Medical Research and the “Guide for the Care and Use of Laboratory Animals” released by the Institute of Laboratory Animal Resources and used by the National Institutes of Health (NIH Publication No. 86-23, revised 1996). All procedures during the study were reviewed and approved by the Ethical Committee of the Land Baden-Württemberg for Animal Experimentation (G-33/18; 30.10.2018).

### 2.2 Experimental groups

**Figure 1:**
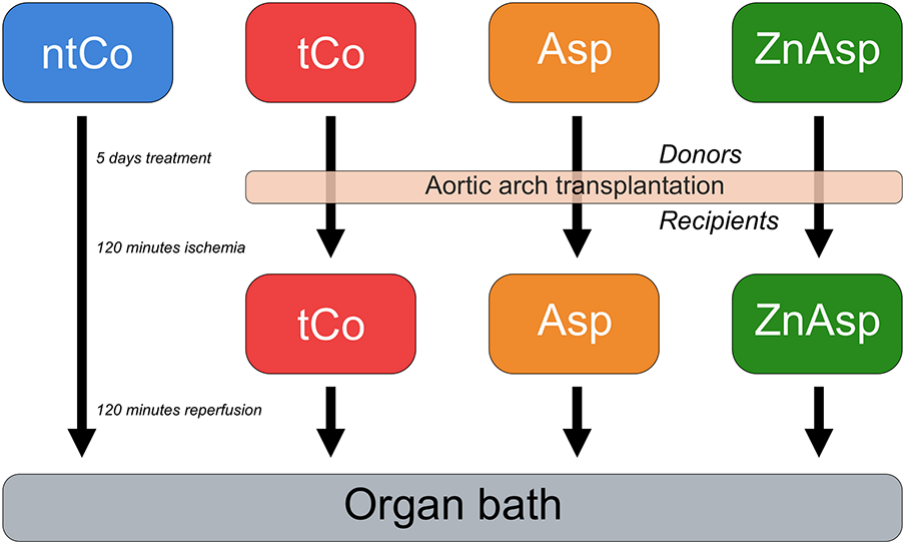
Flowchart of the applied experimental design. ntCo: non-transplanted control group; tCo, transplanted control group; Asp, aspirin group; ZnAsp, zinc-aspirin group.

The rats were randomly divided into the following groups: (1) non-transplanted control group (ntCo, *n* = 8): rats received 1% methylcellulose vehicle, aortic transplantation was not carried out; (2) transplanted control group (tCo, *n* = 8): donor rats received 1% methylcellulose vehicle followed by aortic arch transplantation into the recipient rats (*n* = 8); (3) aspirin group (Asp): donor rats (*n* = 8) were treated with Asp (115 mg/kg), then aortic arches were transplanted into the recipient rats (*n* = 8); (4) zinc-aspirin (ZnAsp) group: donor rats (*n* = 8) were treated with zinc-aspirin (100 mg/kg), with subsequent aortic arch transplantation into the recipient rats (*n* = 8) (Figure 1). As we have hypothesized an add on effect of zinc to the already demonstrated beneficial effect of aspirin in graft protection (10), and previous results have shown that zinc-aspirin complex exerts a more beneficial effect than zinc alone against the endothelial damage-driven neointima formation (13), we have decided not to include a group with zinc treatment alone.

Donor rats and the rats in the ntCo group were treated once daily by oral gavage for 5 days prior to the experiments, while recipient rats received no treatment. Aspirin and zinc-aspirin were suspended in 1% methylcellulose vehicle. The dosage of the applied substances was determined based on literature data (10, 14).

### 2.3 Rat model of heterotopic aortic transplantation

Isogenic Lewis rats were used to avoid organ rejection in the previously reported model of heterotopic aortic transplantation (15).

Briefly, to excise the aortic grafts, we anaesthetized the donor Lewis rats with isoflurane (3% to initiate anaesthesia, 1.75%–2.5% to keep the rats anaesthetized). We also used subcutaneous buprenorphine (0.05–0.1 mg/kg) 45 min before the operation. The donor aortic arch was explanted and flushed using cold physiological saline solution. In the ntCo group, the excised aortic arch was cleaned from fat and connective tissue and were cut into 4 mm-long rings. Two of the rings were freshly mounted for organ bath and the third ring was stored in formalin for further biochemical analysis. In the other groups, aortic arches were harvested and stored in cold physiological saline solution for 80 min. Similarly to the donor rats, recipient rats were anaesthetized and the aortic arches were implanted heterotopically with two end-to-end anastomoses to the abdominal aorta of the recipient rat, to reproduce bypass revascularization. To minimize the variability between experiments, we standardized the duration of cold storage (4°C) at 80 min and implantation at 40 min (120-minute-long ischemic period). The graft was then reperfused with blood *in situ* for 120 min before harvesting it and preparing it for functional measurements or biochemical analysis. After 120 min, recipient rats were sacrificed with an overdose of sodium pentobarbital (150 mg/kg, intraperitoneally).

As part of the procedure, blood flow through the bypass was measured with ultrasonic probe in order to control the quality of the anastomosis. However, we did not quantify the flow as it has not shown any relevance in our previous tests.

### 2.4 *In vitro* organ bath experiments

Functional measurements were carried out on the excised aortic segments, as described previously (15). The harvested vessels were washed with standard Krebs-Henseleit solution, then cleaned of thrombi and tissue remnants before cutting them into 4 mm-long rings and mounting them on hooks in separated organ baths (Radnoti Glass Technology, Monrovia, CA, USA), where they were incubated for 20 min at 37°C in 25 ml/container Krebs-Henseleit solution and aerated with 95% O_2_: 5% CO_2_. After that the aortic rings were placed under a resting tension of 2 g and equilibrated for 60 min.

Prior to every investigation, potassium chloride (KCl, 80 mM) was applied to prepare the aortic rings for stable contractions. Then the aortic rings were rinsed and preconstricted with the alpha-adrenergic receptor agonist phenylephrine (PE, 10^−6^ M) until reaching a stable plateau, followed by adding cumulative concentrations of the endothelium-dependent dilator acetylcholine (ACh, 10^−9^ – 10^−4^ M) for the assessment of relaxation responses. Half-maximum response (EC_50_) values were obtained from individual concentration-responses by fitting experimental data to a sigmoidal equation with the use of Origin 7.0 (Microcal Software, Northampton, MA, USA).

### 2.5 TUNEL reaction

DNA strand breaks were detected using terminal deoxynucleotidyl transferase-mediated dUTP nick-end labeling (TUNEL) assay, as described previously (15). Briefly, the sections were incubated in the dark with TUNEL reaction mixture and 50 µl of Terminal deoxynucleotidyl Transferase (TdT) enzyme for 1 h at 37°C. Afterward, they were washed by phosphate-buffered saline (PBS). The slides were then mounted using 4-,6-diamidino-2-phenylindole (DAPI)-Fluoromount-G™ (SouthernBiotech, Birmingham, AL, USA), covered with a cover glass, and analyzed under a fluorescence microscope. All nuclei were stained blue by DAPI, while the apoptotic cells exhibited fluorescence emission in the red wavelength. For the identification of cell apoptosis rate, we used the Image J (Roche Diagnostics GmbH, Mannheim, Germany) software. Two blinded observers counted the TUNEL-positive cell nuclei, and the mean value was used for the statistical analysis.

### 2.6 Immunohistochemical stainings (CD-31, nitrotyrosine, caspase-3)

The samples were embedded in paraffin, cut into 5-µm thick sections, and stained with hematoxylin eosin to indicate the background. The histological changes of the vessel were detected by targeting specific markers.

Platelet endothelial cell adhesion molecule (PECAM-1) also known as cluster of differentiation 31 (CD-31) is a protein found on the surface of endothelial cells, thus this marker was used to demonstrate the presence of endothelial cells in the lumen of arterial grafts to evaluate the degree of endothelial injury. Following the manufacturer's protocol, anti-CD-31 mouse IgG (Santa Cruz Biotechnology Inc, Heidelberg, Germany) was applied for staining.

Following the previously described method, immunohistochemical staining was performed (MilliporeSigma, Burlington, MA, USA) to identify the nitro-oxidative stress marker nitrotyrosine-3 (NT-3), and mouse monoclonal antibody (Novus Biologicals, Littleton, CO, USA) was used for immunohistochemical localization of caspase-3 to detect the execution phase of cell apoptosis (16).

The samples underwent semi-quantitative immunohistochemical analysis with the use of a conventional light microscope and an imaging software (Olympus, Hamburg, Germany) based on the distribution patterns score multiplied by area score (0–12). Four randomized non-overlapping fields of the aorta were evaluated in a blinded fashion.

### 2.7 Statistical analysis

Data are expressed as means ± standard error of mean (SEM). Shapiro–Wilk test was applied to test the data for normal distribution. The comparison among multiple groups was analyzed using one-way ANOVA and Tukey *post hoc* test, a value of *p* < 0.05 was considered statistically significant. SPSS Statistics 22 (IBM Corp, Armonk, NY, USA) software was used for data analysis.

## 3 Results

### 3.1 Vascular function of aortic rings

**Figure 2:**
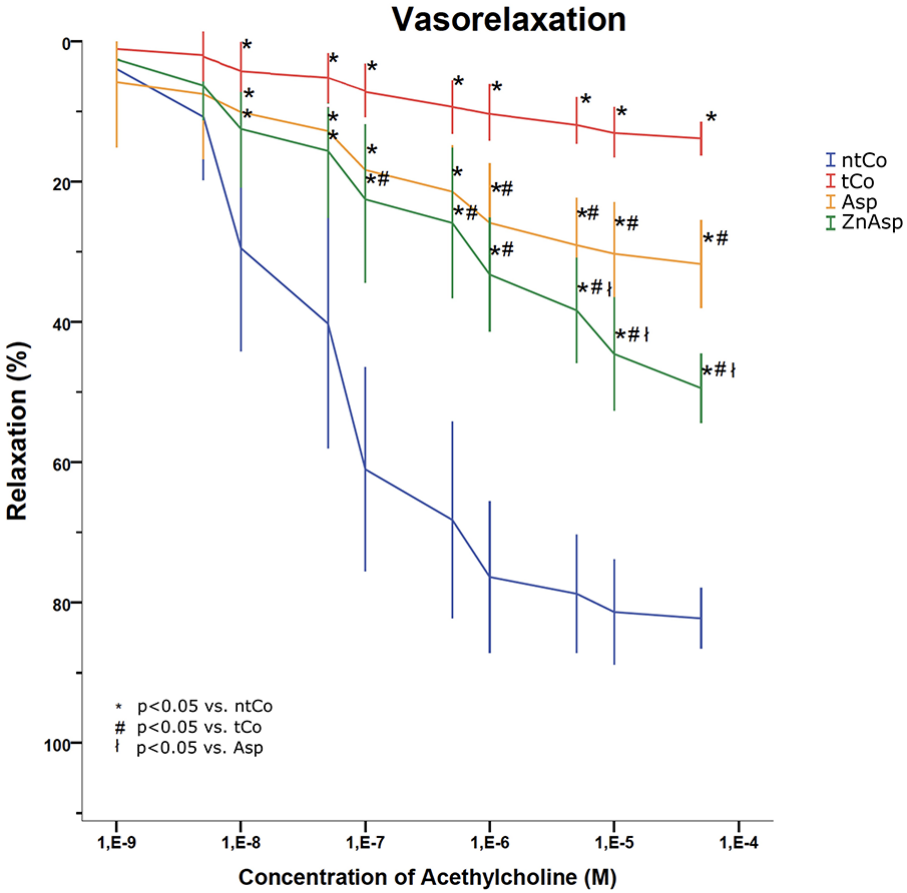
Result of the organ bath functional measurement. Significantly better vasorelaxation activity was found in the aspirin group (Asp) compared to the transplanted control (tCo) group, but zinc-aspirin pretreatment (ZnAsp) resulted in an even better graft protection than treatment with aspirin alone. [*p < 0.05 vs. non-transplanted control group (ntCo), #*p* < 0.05 vs. tCo, ł*p* < 0.05 vs. Asp].

Aortic rings precontracted with phenylephrine (PE) demonstrated a concentration dependent vasorelaxation induced by acetylcholine (Ach) (Figure 2). After 2 h of reperfusion, the transplanted vessels (tCo) showed decreased vasorelaxation compared to the non-transplanted control (ntCo) (14% ± 2% vs. 82% ± 3%, respectively, *p* < 0.05), indicating severe damage. Asp and zinc-aspirin (ZnAsp) both significantly increased the vasorelaxation activity (31% ± 4% and 52% ± 4%, respectively), however zinc-aspirin pretreatment presented higher impact on vasorelaxation compared to the group pretreated with aspirin alone (*p* < 0.05) (Figure 2).

### 3.2 The effect of ischemia-reperfusion injury on arterial graft

**Figure 3:**
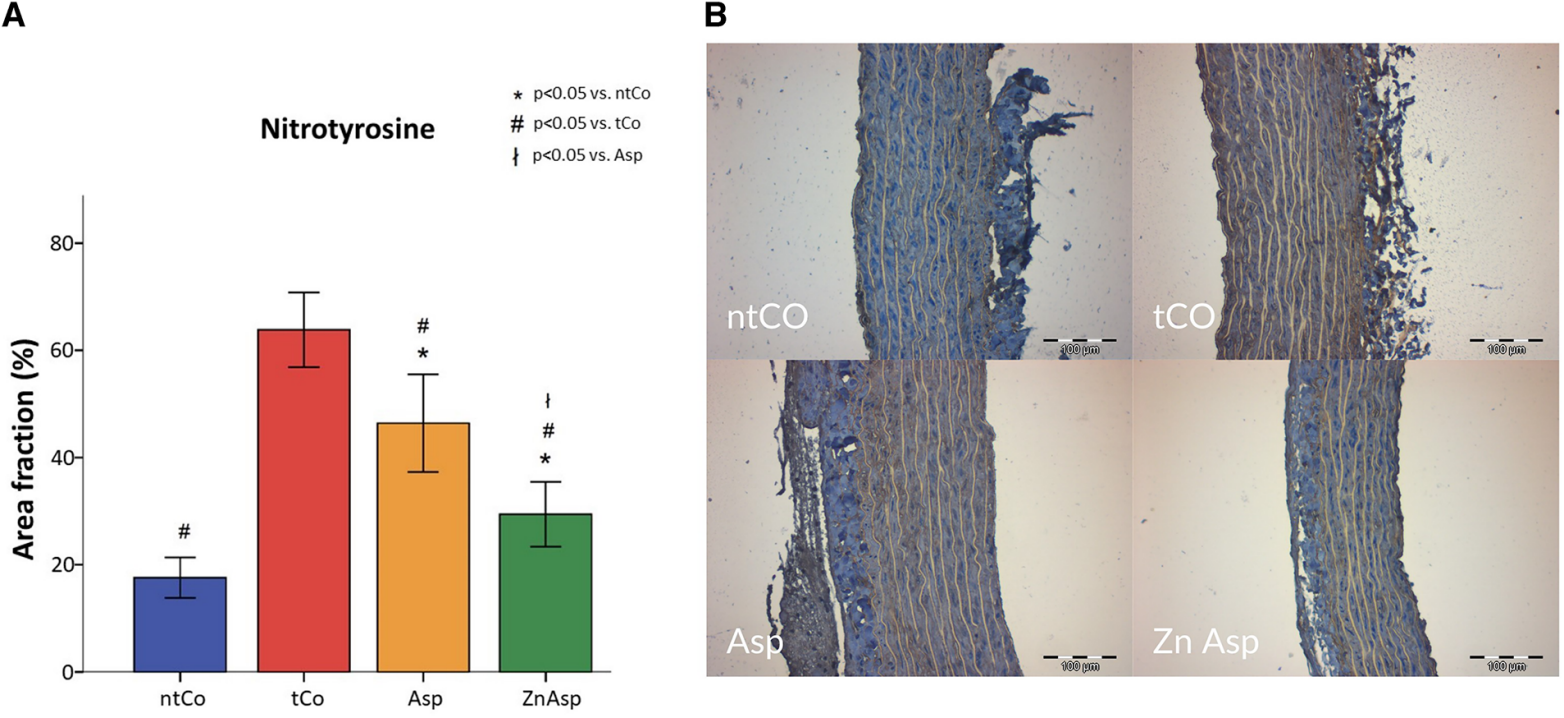
(**A**) aspirin (Asp) significantly decreased the nitro-oxidative stress caused by ischemia-reperfusion injury; however, zinc-aspirin (ZnAsp) showed an even greater protective effect. (*p < 0.05 vs. non-transplanted control group (ntCo), #*p* < 0.05 vs. transplanted control group (tCo), ł*p* < 0.05 vs. Asp). (**B**) The representative immunohistochemistry images demonstrate the expression of nitrotyrosine, shown by the brown color in the aortic wall.

An important factor of ischemia-reperfusion injury is the level of nitro-oxidative stress, which is associated with the amount of nitrotyrosine (NT-3) positive area. The NT-3 immunoreactivity was significantly less increased in the Asp pretreated group after the transplantation than in the tCo group (*p* < 0.05). Zinc-aspirin demonstrated the least amount of nitro-oxidative stress, decreasing it even more effectively than aspirin (*p* < 0.05) (Figure 3).

**Figure 4:**
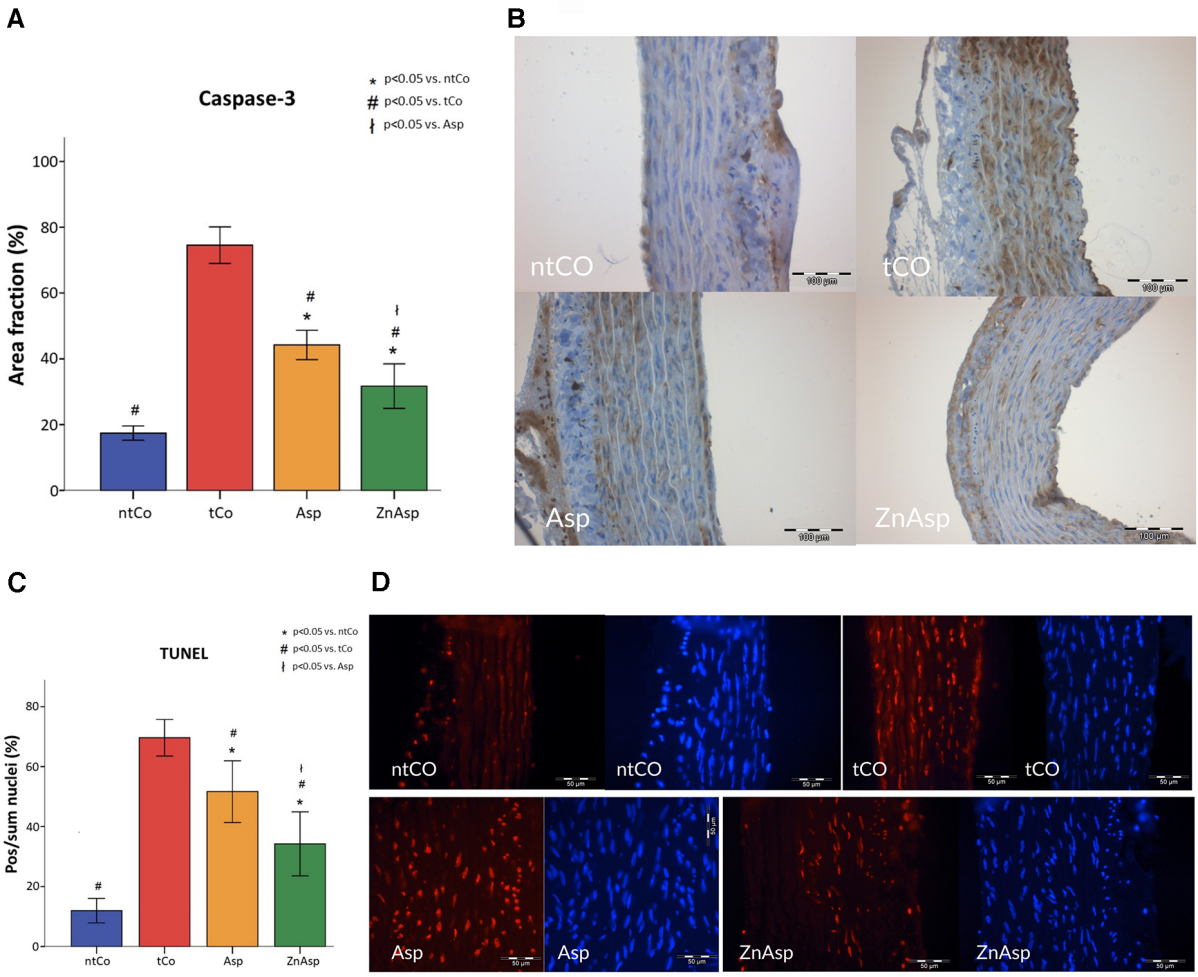
(**A**) caspase-3 immunoreactivity significantly increased following transplantation in transplanted control (tCo) grafts compared to non-transplanted control group (ntCo) as a result of increased apoptosis signaling. This mechanism was prevented by aspirin pretreatment (Asp), however, zinc-aspirin (ZnAsp) demonstrated a significantly better preventive effect. (*p < 0.05 vs. ntCo, #*p* < 0.05 vs. tCo, ł*p* < 0.05 vs. Asp). (**B**) The apoptosis marker caspase-3 is marked with brown color in the representative immunohistochemistry images. (**C**) The TUNEL positive cells were considered as apoptotic cells. Apoptosis inhibition was detected in the aspirin (Asp) and zinc-aspirin (ZnAsp) groups compared to transplanted control group (tCo), however, zinc-aspirin treatment resulted in a significantly decreased apoptosis compared to the Asp group. [*p < 0.05 vs. non-transplanted control group (ntCo), #*p* < 0.05 vs. tCo, ł*p* < 0.05 vs. Asp]. (**D**) Representative micrographs of aortic tissue show red nuclei with fragmented DNA, visualized by TUNEL staining, and blue nuclei representing 4′,6-diamino-2-phenylindole staining.

To study the antiapoptotic effect of the treatments, caspase-3 positivity reflecting apoptotic signaling and terminal deoxynucleotidyl transferase-mediated dUTP nick-end labeling (TUNEL) positivity reflecting apoptotic nuclei were also measured. The tCo group was associated with increased apoptosis demonstrated by increased TUNEL positive nuclei ratio and increased caspase-3 positive area of the sample. Asp pretreatment significantly reduced the apoptosis rate, as it led to a reduced caspase-3 positive area and significantly less DNA strand breaks. The antiapoptotic effect was more pronounced in case of the combined treatment of ZnAsp (*p* < 0.05) (Figure 4).

**Figure 5:**
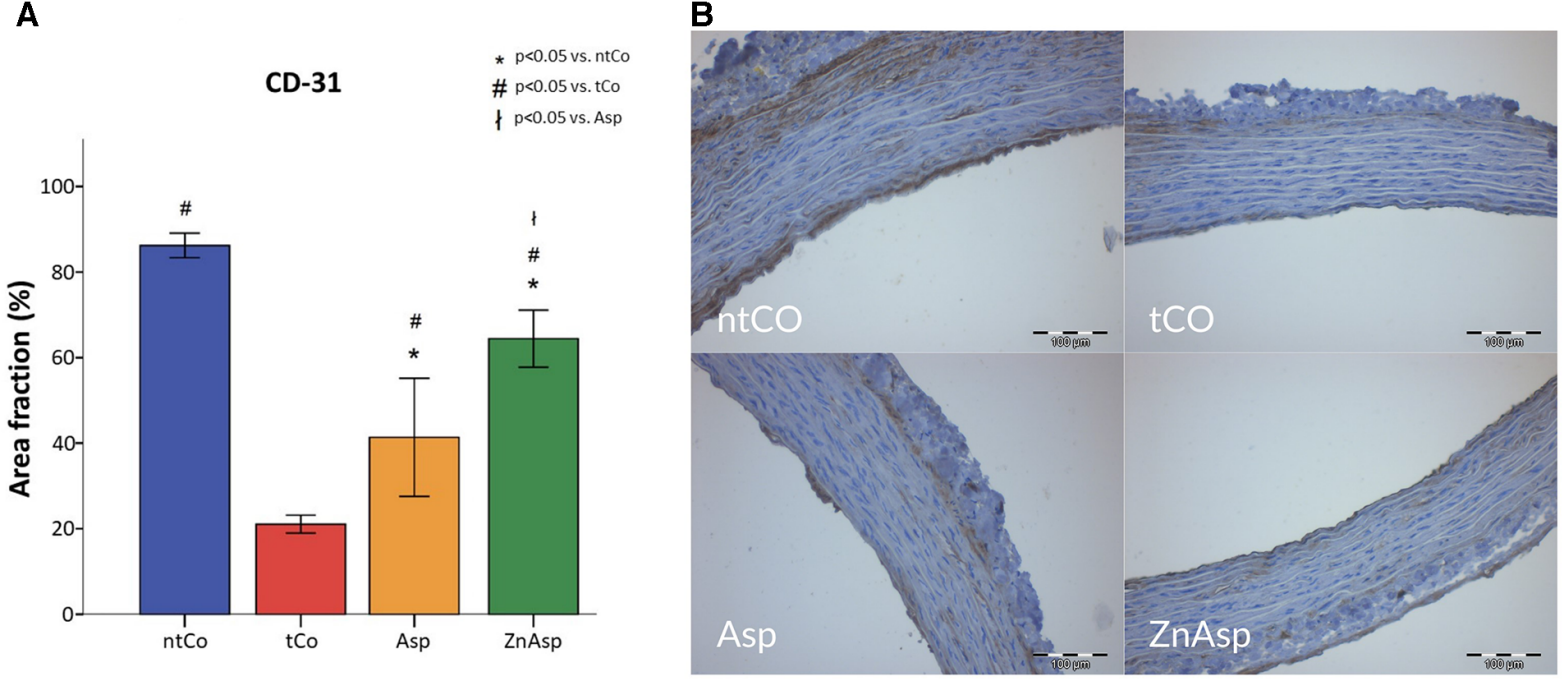
(**A**) CD-31 positive area, reflecting the uninjured endothelium surface was significantly more extensive in the aspirin (Asp) and zinc-aspirin (ZnAsp) groups compared to the transplanted control (tCo) group. Significant difference was also found between the Asp and ZnAsp groups, as zinc-aspirin showed a more intensive endothelium protective effect. [*p < 0.05 vs. non-transplanted control group (ntCo), #*p* < 0.05 vs. tCo, ł*p* < 0.05 vs. Asp]. (**B**) The uninjured endothelium is labelled with brown color in the representative immunohistochemical images.

During aortic transplantation, the graft suffers severe endothelial damage as indicated by the remarkably decreased cluster of differentiation 31 (CD-31) positivity in the tCO group compared to the ntCO group (*p* < 0.05). As shown in Figure 5, aspirin reduced the damage of the internal surface of the graft (*p* < 0.05), and zinc-aspirin demonstrated an even greater protective effect (*p* < 0.05).

## 4 Discussion

In this study, we investigated the protective effects of ZnAsp pretreatment against IRI on free arterial grafts in a rat model of arterial revascularization, and we also compared its effects to that of Asp pretreatment alone. Free arterial grafts without treatment suffered severe endothelial damage on the functional and morphological level. The use of zinc-aspirin prior to the procedure preserved the endothelial function of the grafts and significantly decreased the degree of nitro-oxidative stress and apoptosis. The amount of uninjured endothelium was also significantly higher in the ZnAsp group than in the transplanted control group. The aforementioned findings demonstrate the endothelial protective effect of ZnAsp pretreatment. Preoperative administration of Asp alone exerted the same beneficial effects on the arterial grafts, however, to a significantly lesser extent than ZnAsp treatment, showing the superiority of zinc-aspirin treatment to aspirin alone. As an intact endothelium is a key determinant of graft patency, our results could indicate that zinc-aspirin pretreatment may be able to improve the patency of free arterial grafts after CABG.

The biological properties of ITA enable it to provide the most favorable outcomes in CABG. One of the beneficial components is the increased production of nitric oxide (NO) compared to other conduits (17), which is due to the rich heparin sulfate and endothelial nitric oxide synthase (eNOS) content of the endothelial cells of ITA. NO is an important factor of endothelial homeostasis (18).

Importantly, ITA demonstrates less favorable patency rates when used as a free graft, compared to the *in situ* form (19, 20). However, there are several situations when the use of the free form needs to be preferred. These can be the inadequate length of the *in situ* conduit to reach the target vessel and the injury of the ITA during harvest depending on the site of injury (19). Furthermore, arterial grafts used in addition to the *in situ* ITA are mostly applied as a free arterial graft. The difference between free and *in situ* ITAs is also highlighted by the finding that bilateral ITA grafting demonstrates better results when both ITAs are used as *in situ* graft compared to the setup of applying the second ITA as a free graft (21). Our group has also shown in a porcine model of CABG that endothelial function and integrity were severely impaired in free ITA grafts after reperfusion, however *in situ* grafts showed no signs of injury (17). A major factor leading to poorer outcomes with free grafts is ischemia-reperfusion injury (IRI), which occurs during the harvest and implantation of these conduits.

These observations suggest that finding ways to preserve the function and integrity of free arterial grafts could lead to better results after CABG.

One possible way of graft protection is pharmacological preconditioning, meaning that a drug is administered prior to the procedure with the aim of reducing the expected damage. Our group has identified some promising drugs for this aim in experimental models (8, 9). We have shown in a rat model that treatment with aspirin prior to arterial revascularization significantly reduced the functional and morphological damage suffered by free arterial grafts (10). As many patients take aspirin prior to CABG and there is a debate whether or not this medication should be discontinued before the operation, the findings of our experiment could be clinically highly relevant. Importantly, in agreement with our findings, some studies suggest that the protective effect of aspirin could be further enhanced when using it as a complex with zinc. In a rat model of myocardial injury, preconditioning with zinc and Asp in the form of bis(aspirinato)zinc(II) complex was reported to result in better protection against acute ischemia than pretreatment with aspirin alone. This effect was possibly reached by increasing the level of antioxidant enzymes and the anti-inflammatory cytokine TGF-beta (14). The zinc-complex of aspirin also demonstrated protection against ischemia when it was administered after the occurrence of acute myocardial injury in rats. Increasing the level of antioxidant enzymes was a key element in its mechanism of action (22). Attenuating nitro-oxidative stress and cell apoptosis played an important role in the prevention of diabetic cardiomyopathy in a rat model of type 2 diabetes treated with zinc-aspirin complex (23). Similarly, as an impaired endothelium reduces the patency of the used grafts after CABG, endothelial damage can lead to restenosis in a high number of patients undergoing percutaneous transluminal coronary angioplasty. In a study, balloon injury of the carotid artery was introduced in rats, followed by the treatment of aspirin, zinc or the zinc complex of aspirin. Only zinc-aspirin was found to significantly reduce the ratio of stenosis (13). Thus, our results are consistent with the previous findings that zinc-aspirin treatment is able to exert a protective effect in pathological cardiovascular conditions, presumably mainly by the reduction of oxidative stress. In addition, the zinc complex of aspirin seems to result in better protection than single aspirin treatment.

Furthermore, combined treatment with zinc and aspirin has also been demonstrated to exert beneficial effects in non-cardiovascular settings. In a mouse model of colitis-associated colorectal cancer, the combined treatment of zinc and aspirin showed a protective effect against colitis-associated colorectal cancer by decreasing the degree of inflammation, oxidative stress, cell proliferation and apoptosis (24). In addition, the Bis(aspirinato)Zn complex has been found to be promising in the management of diabetes and metabolic syndrome in spontaneously diabetic mice (25).

Another important aspect of the combined zinc-aspirin treatment is the anti-ulcerogenic activity of zinc (26), which could be beneficial in the prevention of the ulcer-causing side effect of aspirin in case of long-term treatment (27). The continuation of aspirin prior to CABG is still debated due to the increased bleeding risk, however, data suggest its beneficial effects on postoperative outcomes without increasing the rate of surgical re-exploration due to bleeding and the need for red blood cell transfusion when low doses are applied (28). Accordingly, the 2011 American College of Cardiology Foundation/American Heart Association Guidelines for Coronary Artery Bypass Surgery recommends the continuation or administration of aspirin preoperatively or 6 h after the operation (29).

A limitation of our study is the questionable transferability of our findings, as rat aorta differs from human arterial grafts. However, as preconditioning with aspirin has been shown to improve graft patency in humans (30), and zinc seems to be able to enhance the effect of aspirin, it could be expected that treatment with zinc-aspirin complex could be protective for human bypass grafts. Human studies need to be carried out to confirm our findings. A further limitation may be the lack of data on the quantification of blood flow through the grafts. However, at the time of initiating the set-up, we have observed that controlling the flow quality was satisfactory and flow quantification was not required. The above presented data only includes grafts that had sufficient quality of blood flow. Furthermore, acute thrombosis is responsible for early graft failure, however, we did not investigate platelet parameters in this study. As a damaged endothelium is required for the development of acute thrombosis, as well as for the pathological processes that may lead to graft obstruction on the longer term, and we intended to choose end points that may provide information on the possible short- and long-term outcomes, we have focused on the degree of ischemia-reperfusion injury and endothelial damage.

As pretreatment with the zinc complex of aspirin remarkably reduced the functional and morphological damage suffered by free arterial grafts in our study, the administration of zinc-aspirin complex before CABG may be able to improve the patency rate of free arterial grafts, thereby improving the outcomes of the procedure.

## Data Availability

The original contributions presented in the study are included in the article, further inquiries can be directed to the corresponding author.

## References

[1] Melly L Torregrossa G Lee T Jansens JL Puskas JD. Fifty years of coronary artery bypass grafting. J Thorac Dis. (2018) 10(3):1960–7. 10.21037/jtd.2018.02.43 29707352 PMC5906252

[2] Gaudino M Antoniades C Benedetto U Deb S Di Franco A Di Giammarco G ATLANTIC (arterial grafting international consortium) alliance. Mechanisms, consequences, and prevention of coronary graft failure. Circulation. (2017) 136(18):1749–64. 10.1161/CIRCULATIONAHA.117.027597 29084780

[3] Korkmaz-Icöz S Ballikaya B Soethoff J Kraft P Sayour AA Radovits T Graft preservation solution DuraGraft® alleviates vascular dysfunction following in vitro ischemia/reperfusion injury in rats. Pharmaceuticals. (2021) 14(10):1028. 10.3390/ph14101028 34681252 PMC8538682

[4] Cowled P Fitridge R. Pathophysiology of reperfusion injury. In: Fitridge R Thompson M, editors. Mechanisms of Vascular Disease: A Reference Book for Vascular Specialists. Adelaide, AU: University of Adelaide Press (2011). p. 18. 30484990

[5] Dion R Glineur D Derouck D Verhelst R Noirhomme P El Khoury G Long-term clinical and angiographic follow-up of sequential internal thoracic artery grafting. Eur J Cardiothorac Surg. (2000) 17(4):407–14. 10.1016/S1010-7940(00)00370-5 10773563

[6] Veres G Hegedűs P Barnucz E Zöller R Klein S Radovits T Graft preservation with heparinized blood/saline solution induces severe graft dysfunction. Interact Cardiovasc Thorac Surg. (2015) 20(5):594–600. 10.1093/icvts/ivv010 25672335

[7] Elbadawi A Saad M Nairooz R. Aspirin use prior to coronary artery bypass grafting surgery: a systematic review. Curr Cardiol Rep. (2017) 19(2):18. 10.1007/s11886-017-0822-5 28213669

[8] Veres G Bai Y Stark KA Schmidt H Radovits T Loganathan S Pharmacological activation of soluble guanylate cyclase improves vascular graft function. Interact Cardiovasc Thorac Surg. (2021) 32(5):803–11. 10.1093/icvts/ivaa329 33515043 PMC8923409

[9] Veres G Hagenhoff M Schmidt H Radovits T Loganathan S Bai Y Targeting phosphodiesterase-5 by vardenafil improves vascular graft function. Eur J Vasc Endovasc Surg. (2018) 56(2):256–63. 10.1016/j.ejvs.2018.03.025 29724533

[10] Veres G Benke K Stengl R Bai Y Stark KA Sayour AA Aspirin reduces ischemia-reperfusion injury induced endothelial cell damage of arterial grafts in a rodent model. Antioxidants. (2022) 11(2):177. 10.3390/antiox11020177 35204060 PMC8868254

[11] Vane JR Botting RM. The mechanism of action of aspirin. Thromb Res. (2003) 110(5-6):255–8. 10.1016/S0049-3848(03)00379-7 14592543

[12] Akbari G. Role of zinc supplementation on ischemia/reperfusion injury in Various organs. Biol Trace Elem Res. (2020) 196(1):1–9. 10.1007/s12011-019-01892-3 31828721

[13] Hegedűs P Korkmaz S Radovits T Schmidt H Li S Yoshikawa Y Bis (aspirinato) zinc (II) complex successfully inhibits carotid arterial neointima formation after balloon-injury in rats. Cardiovasc Drugs Ther. (2014) 28(6):533–9. 10.1007/s10557-014-6549-2 25129612

[14] Korkmaz S Atmanli A Li S Radovits T Hegedűs P Barnucz E Superiority of zinc complex of acetylsalicylic acid to acetylsalicylic acid in preventing postischemic myocardial dysfunction. Exp Biol Med. (2015) 240(9):1247–55. 10.1177/1535370215570184 PMC4935359 25670850

[15] Veres G Hegedűs P Barnucz E Schmidt H Radovits T Zöller R Tiprotec preserves endothelial function in a rat model. J Surg Res. (2016) 200(1):346–55. 10.1016/j.jss.2015.06.062 26219206

[16] Liaudet L Soriano FG Szabó E Virág L Mabley JG Salzman AL Protection against hemorrhagic shock in mice genetically deficient in poly(ADP-ribose)polymerase. Proc Natl Acad Sci. (2000) 97(18):10203–8. 10.1073/pnas.170226797 10954738 PMC27808

[17] Veres G Schmidt H Hegedűs P Korkmaz-Icöz S Radovits T Loganathan S Is internal thoracic artery resistant to reperfusion injury? Evaluation of the storage of free internal thoracic artery grafts. J Thorac Cardiovasc Surg. (2018) 156(4):1460–9. 10.1016/j.jtcvs.2018.05.079 30257283

[18] Otsuka F Yahagi K Sakakura K Virmani R. Why is the mammary artery so special and what protects it from atherosclerosis? Ann Cardiothorac Surg. (2013) 2(4):519–26. 10.3978/j.issn.2225-319X.2013.07.06 23977631 PMC3741888

[19] Ranney DN Williams JB Mulder H Wojdyla D Cox ML Gibson CM Comparison of outcomes and frequency of graft failure with use of free versus in situ internal mammary artery bypass conduits (from the PREVENT IV trial). Am J Cardiol. (2019) 123(4):571–5. 10.1016/j.amjcard.2018.11.029 30538035 PMC9757022

[20] Verhelst R Etienne PY El Khoury G Noirhomme P Rubay J Dion R. Free internal mammary artery graft in myocardial revascularization. Cardiovasc Surg. (1996) 4(2):212–6. 10.1016/0967-2109(96)82318-0 8861440

[21] Marzouk M Kalavrouziotis D Grazioli V Meneas C Nader J Simard S Long-term outcome of the *in situ* versus free internal thoracic artery as the second arterial graft. J Thorac Cardiovasc Surg. (2021) 162(6):1744–52.e7. 10.1016/j.jtcvs.2020.03.003 32305200

[22] Korkmaz-Icöz S Atmanli A Radovits T Li S Hegedüs P Ruppert M Administration of zinc complex of acetylsalicylic acid after the onset of myocardial injury protects the heart by upregulation of antioxidant enzymes. J Physiol Sci. (2016) 66(2):113–25. 10.1007/s12576-015-0403-6 26497333 PMC10717564

[23] Korkmaz-Icöz S Al Said S Radovits T Li S Brune M Hegedűs P Oral treatment with a zinc complex of acetylsalicylic acid prevents diabetic cardiomyopathy in a rat model of type-2 diabetes: activation of the akt pathway. Cardiovasc Diabetol. (2016) 15:75. 10.1186/s12933-016-0383-8 27153943 PMC4858866

[24] Babu SSN Singla S Jena G. Role of combination treatment of aspirin and zinc in DMH-DSS-induced colon inflammation, oxidative stress and tumour progression in male BALB/c mice. Biol Trace Elem Res. (2023) 201(3):1327–43. 10.1007/s12011-022-03241-3 35438409

[25] Yoshikawa Y Adachi Y Yasui H Hattori M Sakurai H. Oral administration of Bis(aspirinato)zinc(II) complex ameliorates hyperglycemia and metabolic syndrome-like disorders in spontaneously diabetic KK-A(y) mice: structure-activity relationship on zinc-salicylate complexes. Chem Pharm Bull. (2011) 59(8):972–7. 10.1248/cpb.59.972 21804241

[26] Opoka W Adamek D Plonka M Reczynski W Bas B Drozdowicz D Importance of luminal and mucosal zinc in the mechanism of experimental gastric ulcer healing. J Physiol Pharmacol. (2010) 61(5):581–91. 21081802

[27] Cryer B Mahaffey KW. Gastrointestinal ulcers, role of aspirin, and clinical outcomes: pathobiology, diagnosis, and treatment. J Multidiscip Healthc. (2014) 7:137–46. 10.2147/JMDH.S54324 24741318 PMC3970722

[28] Aboul-Hassan SS Stankowski T Marczak J Peksa M Nawotka M Stanislawski R The use of preoperative aspirin in cardiac surgery: a systematic review and meta-analysis. J Card Surg. (2017) 32(12):758–74. 10.1111/jocs.13250 29205497

[29] Hillis LD Smith PK Anderson JL Bittl JA Bridges CR Byrne JG 2011 ACCF/AHA guideline for coronary artery bypass graft surgery: executive summary: a report of the American college of cardiology foundation/American heart association task force on practice guidelines. Circulation. (2011) 124(23):2610–42. Epub 2011 Nov 7. Erratum in: Circulation. 2011 Dec 20;124(25):e956. Erratum in: Circulation. 2012 Aug 14;126(7):e105. 10.1161/CIR.0b013e31823b5fee 22064600

[30] Bybee KA Powell BD Valeti U Rosales AG Kopecky SL Mullany C Preoperative aspirin therapy is associated with improved postoperative outcomes in patients undergoing coronary artery bypass grafting. Circulation. (2005) 112(9 Suppl):I286–92. 10.1161/CIRCULATIONAHA.104.522805 16159833

